# Modeling Single Ventricle Physiology: Review of Engineering Tools to Study First Stage Palliation of Hypoplastic Left Heart Syndrome

**DOI:** 10.3389/fped.2013.00031

**Published:** 2013-10-30

**Authors:** Giovanni Biglino, Alessandro Giardini, Tain-Yen Hsia, Richard Figliola, Andrew M. Taylor, Silvia Schievano, MOCHA Collaborative Group

**Affiliations:** ^1^Centre for Cardiovascular Imaging, UCL Institute of Cardiovascular Science, London, UK; ^2^Cardiorespiratory Unit, Great Ormond Street Hospital for Children, NHS Foundation Trust, London, UK; ^3^Departments of Bioengineering and Mechanical Engineering, Clemson University, Clemson, SC, USA

**Keywords:** Norwood procedure, single ventricle, shunting, computational modeling, experimental modeling

## Abstract

First stage palliation of hypoplastic left heart syndrome, i.e., the Norwood operation, results in a complex physiological arrangement, involving different shunting options (modified Blalock-Taussig, RV-PA conduit, central shunt from the ascending aorta) and enlargement of the hypoplastic ascending aorta. Engineering techniques, both computational and experimental, can aid in the understanding of the Norwood physiology and their correct implementation can potentially lead to refinement of the decision-making process, by means of patient-specific simulations. This paper presents some of the available tools that can corroborate clinical evidence by providing detailed insight into the fluid dynamics of the Norwood circulation as well as alternative surgical scenarios (i.e., virtual surgery). Patient-specific anatomies can be manufactured by means of rapid prototyping and such models can be inserted in experimental set-ups (mock circulatory loops) that can provide a valuable source of validation data as well as hydrodynamic information. Such models can be tuned to respond to differing the patient physiologies. Experimental set-ups can also be compatible with visualization techniques, like particle image velocimetry and cardiovascular magnetic resonance, further adding to the knowledge of the local fluid dynamics. Multi-scale computational models include detailed three-dimensional (3D) anatomical information coupled to a lumped parameter network representing the remainder of the circulation. These models output both overall hemodynamic parameters while also enabling to investigate the local fluid dynamics of the aortic arch or the shunt. As an alternative, pure lumped parameter models can also be employed to model Stage 1 palliation, taking advantage of a much lower computational cost, albeit missing the 3D anatomical component. Finally, analytical techniques, such as wave intensity analysis, can be employed to study the Norwood physiology, providing a mechanistic perspective on the ventriculo-arterial coupling for this specific surgical scenario.

## Introduction

Hypoplastic left heart syndrome (HLHS) is a form of single ventricle physiology characterized by a rudimentary, non-functional, or absent left ventricle, and by a consequent in-parallel arrangement of the systemic and pulmonary circulations (1). This condition requires a complex, staged surgical palliation in order to allow appropriate blood oxygenation and patient’s survival (2). Diagnosed *in utero* (2), HLHS is tackled at birth, with the first stage of palliation, namely the Norwood procedure (3), being performed in the first days of life. The Norwood operation entails in fact providing a source of pulmonary blood flow following the natural closure of the ductus arteriosus after birth, while also enlarging the otherwise hypoplastic ascending aorta. Delivery of blood flow to the pulmonary circulation is achieved by means of shunting, with substantially different options currently available, including:

- Modified Blalock-Taussig (mBT) shunt (4) from the innominate artery to the right pulmonary artery.
- Sano shunt from the right ventricle to the main pulmonary artery (RV-PA conduit), employed in the so-called Sano modification of the Norwood procedure (5).
- Central shunt from the ascending aorta to the pulmonary arteries (6).

**Figure 1:**
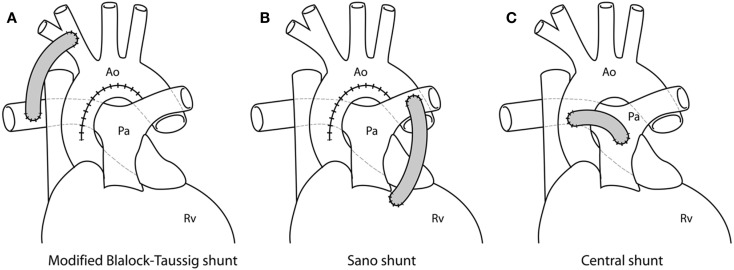
**Different shunting options for first stage palliation of HLHS, shown from idealized drawings: (A) modified Blalock-Taussig shunt from the innominate artery to the right pulmonary artery; (B) Sano shunt from the right ventricle (Rv) to the pulmonary artery (Pa); (C) central shunt from the ascending aorta (Ao) to the pulmonary artery**.

Examples of different shunts are shown in Figure 1.

With regard to aortic arch reconstruction, the surgery involves enlarging the ascending aorta using a patch, typically either homograft or bovine pericardium (7, 8).

The physiology resulting from the Norwood operation is clearly very complex and, albeit just a transition to second stage of palliation occurring around the sixth month of life (2), it can lead to complications. Complications after the Norwood operation are still common, with a high mortality risk (9). While some of the complications have been linked with pre-operative characteristics, such as patient’s weight or pre-operative mechanical ventilator/circulatory support (9), other variables are linked to the actual surgery. The choice of shunt type, for instance, can depend on the surgeon’s own expertise and judgment, as well as on a center’s preference, but there are established hemodynamic differences between shunts which have been discussed in the clinical literature (10–12). Clinical investigations, however, have not been conclusive with regard to variables such as shunt size, shunt placement, or extent of surgical arch reconstruction. In other words, there is still potential for refining and optimizing the hemodynamics following the Norwood procedure.

While clinical investigations provide the necessary data on the outcomes to ultimately evaluate, for example, differences between shunt type, further insight into the physiology and opportunity for in-depth tests on specific variables can be gained by means of engineering modeling tools. In the context of studying congenital heart disease, modeling tools can provide:

- access to data that is difficult to acquire in the clinical environment (e.g., coronary perfusion data)
- detailed local fluid dynamics information
- a test bed for parametric studies
- a controllable and reproducible environment for hemodynamic investigations
- a source of alternative/virtual scenarios for treatment options
- a setting for evaluation of devices, where needed
- a tool for education and development
- a tool for dissemination.

Different models can be constructed, depending on the purpose of the study, but in general these can be categorized into three main groups: experimental (*in vitro* set-ups), computational (*in silico* simulations), and analytical (purely mathematical models). This Review will briefly describe, for each of these categories, some of the models that have been proposed in order to address issues related to the Norwood procedure and their relevant findings, aiming to highlight those variables most likely to impact the hemodynamics of Stage 1 circulation and appreciating the importance of factors such as the concomitant presence of other complications (e.g., aortic coarctation), including some methodological considerations.

It should be noted that a less surgically invasive approach to Stage 1 palliation has been introduced in recent years, indicated as the “hybrid” Norwood (13), characterized by stenting of the ductus arteriosus and banding of branch pulmonary arteries. This will not be discussed in this Review, as it is described in greater detail in another article of this Special Issue.

## Experimental Models

Cardiovascular experimental models, in general, can be particularly informative for device testing (e.g., fatigue evaluation, device migration) (14, 15) and, importantly, can represent a source of reproducible “real world data” for validation of computational models (16). These models usually take the form of mock circulatory loops, whose level of complexity may vary depending on the purpose of the experiment (17), from rather simple rigs with lumped resistive and compliant elements (18) or systems incorporating some anatomical realistic elements (19) to circuits including the effect of respiration (20) and full circulatory mock loops with all main vascular components (21).

In the context of investigating the Norwood physiology or some of the variables affecting it, one experimental study (22) employed a pulsatile flow model including a pulsatile flow generator, and parallel systemic and pulmonary vasculatures connected by aorto-pulmonary shunts. The system was used to test a range of BT shunt lengths and diameters, and ultimately to verify the relation between Doppler-predicted pressure gradient and the pressure gradient measured in actual Gore-Tex^®^ shunts placed in the circuit. Results showed that Doppler estimates of pressure gradients approach true measurements only in cases of large shunts, whereas for BT shunts with diameter <5 mm the simplified Bernoulli equation used for Doppler underestimates such gradient. A later study employing similar methodology (23) expanded these observations, whereby three-dimensional (3D) models of mBT shunts with and without stenosis were also tested experimentally. This study showed that the Doppler-measured gradients underestimated catheter-measured gradients in mBT shunts with diffuse stenosis, while in other scenarios (no stenosis, outlet stenosis, inlet stenosis), the Doppler pressure gradients showed underestimation of catheter measures at low gradients and improved estimation at higher gradients. Studies of this nature can have the benefit of informing on the nature and the reliability of clinical measurements, demonstrating potential pitfalls of accepted approximations (e.g., Bernoulli equation for pressure drop estimate).

Another *in vitro* study (24) focused on pressure-flow relationships in mBT shunts taking into account anastomotic distensibility and restrictions due to the presence of sutures, whereby two actual Gore-Tex^®^ shunts (3 and 4 mm diameter) were tested in a hydraulic circuit under a range of steady flow rates and pulmonary pressures. It was shown that pressure-flow relationship was affected by changes in pulmonary artery pressure, especially at the distal site; however the total pressure drop did not change substantially. This study suggested that the effect of afterload pressure on mBT shunt pressure-flow relationship is not determinant, while area reduction at the anastomoses sites due to suturing should be taken into account. Vascular resistance-flow relationship in an mBT shunt scenario was further investigated *in vitro* using a set-up constructed from sheep blood vessels (25), generating pulsatile flow by means of a ventricular assist device and testing a range of pulmonary vascular resistances.

**Figure 2:**
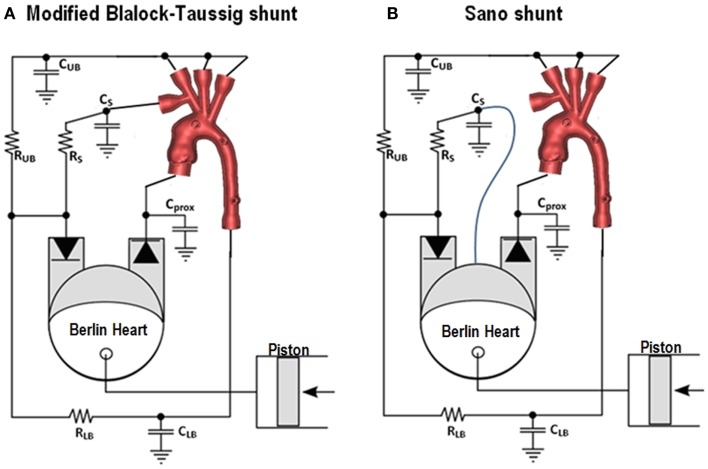
**Experimental set-ups for simulating the circulation following the Norwood operation, with modified Blalock-Taussig shunt (A) and Sano shunt (B)**. The mock loops include a 3D patient-specific anatomical model. The Berlin Heart EXCOR simulates the single ventricle. C_UB_ = lumped compliance for upper body district, R_UB_ = lumped resistance for upper body district, C_LB_ = lumped compliance for lower body district, R_LB_ = lumped resistance for lower body district, C_S_ = lumped compliance for pulmonary district, R_S_ = lumped resistance for pulmonary district, C_prox_ = proximal compliance compensating for rigid 3D model.

Other studies adopted a patient-specific approach to the experimental investigation of this physiology. A mock circulatory system involving 3D patient-specific anatomical models was shown to behave in a physiological range for both mBT and Sano shunts settings (26, 27). The patient-specific models were reconstructed from cardiovascular magnetic resonance (CMR) data (28) and printed with transparent rigid resins using rapid prototyping technology. These studies adopt a multi-scale approach (29), in the sense that they combine a 3D anatomical section with a lumped parameter network (LPN) representing the remainder of the circulation. In both cases pulsatile flow was generated with a pediatric Berlin Heart EXCOR ventricular assist device. The mBT shunt was simulated by a conduit positioned from the innominate artery of the 3D model and the pulmonary section of the circuit. The Sano shunt was simulated by a connection from the de-airing valve of the Berlin Heart (simulating the ventricular anastomosis) to the pulmonary section of the circuit. These arrangements are shown schematically in Figure 2. The usefulness of including patient-specific models is that it allows to measure parameters, e.g., pressure drop across a coarctation, using real geometries. These experimental set-ups allow both for parametric studies as well as tuning to patient-specific hemodynamic values derived from clinical data, depending on the purpose of the study, and they can be compatible with visualization techniques, as discussed in the following section.

## Imaging Techniques

**Figure 3:**
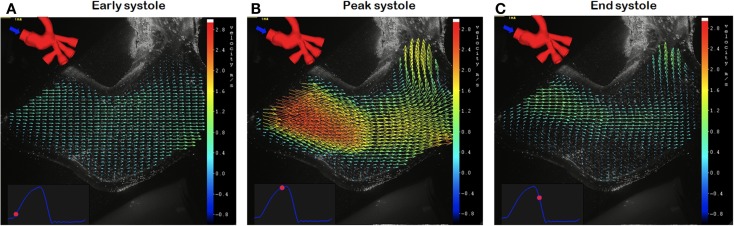
**Example of particle image velocimetry (PIV) data, obtained in a patient-specific anatomical model of Stage 1 physiology, showing velocity vectors at early (A), peak (B) and end (C) systole**.

Experimental set-ups of Norwood physiology can be adapted so to be compatible with visualization techniques, in order to gather further insight into the local fluid dynamics. One technique that has been extensively used *in vitro* for hemodynamic studies is particle image velocimetry (PIV), especially for valve testing (30–32). PIV is an optical technique providing accurate quantitative measurement of instantaneous velocity flow fields across a plane, by means of illuminating a surface with a laser sheet and seeding the fluid with particles (“tracers”) whose movement is recorded by a high-speed camera (33). A study employing a model of Norwood physiology with 3D anatomical components (26) has shown the applicability of PIV acquisition within this context (34). This preliminary study, which involves tuning the circuit to patient-specific values derived from clinical data, presents the velocity vector information (Figure 3) that can be derived by using the PIV technique even with small aortic models of Norwood patients.

Another visualization technique that can be potentially adapted for experimental studies is 4D CMR (35). This method, which has been greatly improved in recent years and whose capabilities have been and are being explored in a wide range of clinical studies, provides exquisite imaging data. The advantages of using this method in the clinical assessment of HLHS patients have been discussed, especially at the stage of complete Fontan circulation (36). However, experimental applications directly focused on the Norwood procedure have not been tested yet. Experimental studies involving 4D CMR are few and have focused on the assessment of a ventricular assist device (37) and on the fluid dynamics in the ascending aorta following repair of transposition of the great arteries (38). The latter study showed how 4D CMR acquisitions can be performed with a CMR-compatible mock loop including 3D patient-specific models, suggesting the potential for using this technique in models of Stage 1 physiology, although the small dimensions of the vessels at the time of the Norwood procedure could pose a concern in terms of spatial resolution. This application warrants further study.

## Computational Models

Computational models of the Norwood physiology have been explored and improved in the past 15 years.

Earlier studies employed LPN models of the Norwood circulation (39, 40). These studies focused on global fluid dynamics and oxygen transport characteristics, but failed to describe local fluid dynamics and the influence of variables related to the shunts, e.g., shunt positioning.

A later study showed how a LPN model of the circulation can be coupled with a detailed 3D model of the shunt, using a multi-scale approach to prescribe appropriate boundary conditions for the 3D models of the Norwood circulation (41). This study aimed to compare coronary and pulmonary blood flows in a central shunt vs. mBT shunt configuration, considering three shunt sizes (3, 3.5, and 4 mm diameter). Results showed that the average shunt flow rate is higher for the central shunt option. As expected, pulmonary flow increased with shunt size for both options. It was also indicated that the central shunt tends to favor perfusion to the right lung, while the mBT shunt tends to favor the left lung. Finally, a smaller percentage of aortic flow is distributed to the coronary circulation in the presence of a central shunt, suggesting a potential effect on ventricular function. These observations were expanded in another study (42) which included 3D models of mBT and central shunts (3, 3.5, and 4 mm diameter) vs. Sano shunts (4, 5, and 6 mm diameter). The hydraulic lumped resistances, compliances, inertances, and elastances representing the systemic, coronary, and pulmonary circulations and the heart were identical in the two models, essentially isolating the effect of different shunt type. Again, a multi-scale approach was used to couple the 3D models with the LPN. Higher aortic diastolic pressure, decreased pulmonary arterial pressure, reduced pulmonary-to-systemic flow ratio, and higher coronary perfusion pressure were measured in the Sano configuration. Also, a minimal regurgitant flow was noted in the Sano conduit. Computer simulation results were in good agreement with post-operative catheterization data, supporting the use of mathematical modeling in the study of Norwood physiology. This study pointed out that, from a computational perspective, the use of a multi-scale approach is “mandatory.”

Computational fluid dynamics (CFD) have been used to analyze blood flow in a Norwood anatomy derived from computed tomography (CT) datasets (43). This study presented a case-study of a complex case of congenital heart disease (HLHS palliated with Sano modification of the Norwood procedure, aortic stenosis, hypoplastic aortic arch, coarctation of the aorta, and ventricular septal defect). Information such as pressure and wall shear stress distribution on the vessel wall as well as velocity vectors and streamlines can be obtained from these simulations. The authors concluded that such a computational hemodynamic system can quantitatively estimate the quality of congenital heart disease surgery. Albeit this point may be arguable, especially based on a single case-study and on the lack of biological phenomena in this type of modeling, it is undeniable that a large amount of valuable information can be extrapolated from CFD models for the purpose of informing, if not estimating, this type of complex surgery.

Patient-specific computational simulations were performed in nine patients in order to evaluate different types of Norwood arch reconstructions and to assess the cardiac workload on the single ventricle (44). This paper included cases of aortic atresia and aortic stenosis. Results, including quantities of energy loss and wall shear stress, suggested that the quality of arch reconstruction (e.g., smooth arch angle) is important for reducing the cardiac workload. Energetic efficiency is difficult to measure clinically and computational simulations can provide insight into such valuable measures.

**Figure 4:**
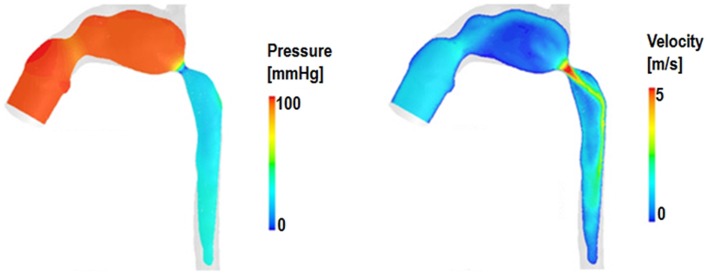
**Example of pressure and velocity maps in the 3D domain of a multi-scale simulation of HLHS post Stage 1 including a significant aortic coarctation, highlighting features such as pressure drop across the aortic narrowing as well as the velocity jet across the coarctation itself**.

An example of hemodynamic information (pressure and velocity data) extracted from a multi-scale model of HLHS following Stage 1 palliation including aortic coarctation is shown in Figure 4.

Computational techniques can also include optimization algorithms and a recent study has employed a closed loop multi-scale model (including an idealized mBT shunt 3D component) integrated with a fully automated derivative-free optimization algorithm to assess optimal shunt configuration in terms of (a) shunt diameter, (b) location of anastomosis, and (c) shunt angle (45). Results showed that shunt diameter affects changes in oxygen delivery the most, but shunt positioning does also influence such changes, and these data showed that coronary artery flow is directly related to shunt position. Small shunt diameter with proximal shunt-brachiocephalic anastomosis was optimal for systemic oxygen delivery, while large shunt diameter with a distal anastomosis was optimal for coronary oxygen delivery.

**Figure 5:**
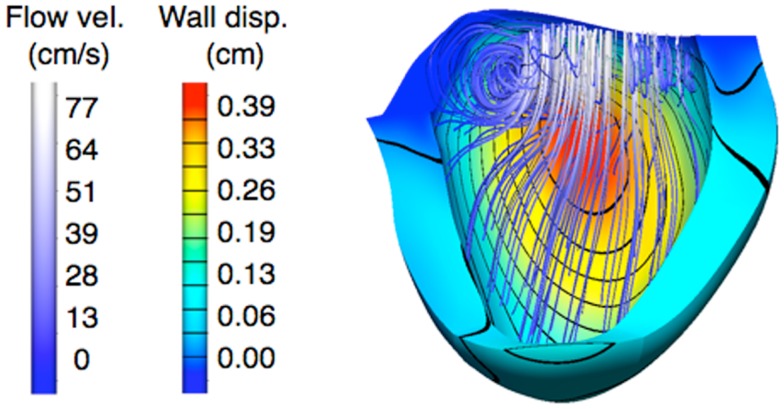
**Example of results from fluid structure interaction (FSI) simulations, providing information on displacement of the myocardium, as well as showing vortex formation and blood streamlines in a patient after Stage 1 palliation**.

All the abovementioned studies assumed rigid blood vessel walls. In other cases, it is crucial to include so-called fluid structure interaction (FSI) phenomena in the simulations. One good example in the context of Norwood physiology is a study in which numeric simulations were performed to investigate the interaction between blood flow and myocardial motion during diastole (46). More specifically, the effect of ventricular cavity shape and tricuspid inflow topology were evaluated in four patients’ anatomies, with regard to filling dynamics and assessment of diastolic function in patients post Stage 1 surgery. It was observed that both these parameters (i.e., inflow topology and cavity shape) affect vortex ring formation, thus influencing intra-ventricular pressure gradients and flow dynamics inside the single ventricle. Differences between patients in terms of myocardial displacements can be well appreciated from the FSI modeling results (Figure 5).

Computational methodologies have recently also been used to study the hybrid Norwood procedure (47, 48).

## Analytical Tools: Wave Intensity Analysis

Further insight into the Norwood physiology can be gained by means of analytical methods, such as wave intensity analysis. Wave intensity is a hemodynamic index evaluating the working condition of the heart in relation to the rest of the vasculature and, as such, it provides information on ventriculo-arterial (VA) coupling (49). Literature on wave intensity analysis has shown its potential in investigating VA coupling in different scenarios, such as the fetal circulation (50) or healthy adults (51). Traditionally necessitating invasive pressure and velocity acquisitions, wave intensity analysis can nowadays be performed non-invasively, based on CMR (52). This technique allows semi-automatic and retrospective analysis on routinely acquired phase-contrast CMR datasets, and it has been applied to a group of HLHS patients to evaluate the effect of surgical arch reconstruction on VA coupling (53). Based on clinical observations that report stiffening of the surgically enlarged aortic arch (54, 55), thus likely increasing the impedance to ventricular ejection, this study compared single ventricle patients with and without arch reconstruction. Based on wave intensity parameters previously identified as possible surrogates for ventricular function (56), results highlighted that VA coupling is likely to be compromised in patients with surgical reconstruction. This appeared to be linked to two main variables, i.e., the size of the patch and the stiffness of the patch. While this study was carried out in a small population (21 subjects in total) and larger studies are needed to infer the clinical relevance of these observations, it showed the potential of CMR-based wave intensity analysis in providing additional knowledge about the Norwood physiology.

## Advantages and Disadvantages of Different Techniques

Compared to *in vivo* data, both experimental (*in vitro*) and computational (*in silico*) techniques present the advantage of creating reproducible and controllable environments suitable for performing parametric studies and for acquiring data systematically.

One advantage of experimental models is represented by their natural 3D nature and tactile component, which can have educational and communication benefits as well as allowing for physically implanting devices that need testing (57). Furthermore, experimental models naturally take into account FSI phenomena, whereby a suitably designed compliant phantom could represent a good approximation of a blood vessel (58). However, setting up an *in vitro* experiment – or repeating measurements on different phantoms – can be time consuming.

Imaging techniques can be extremely informative. Applications of PIV are confined to the research arena. The feasibility of PIV measurements with a Norwood anatomy has been shown (34), however several considerations inherent to the PIV set-up (e.g., matching the refractive index of the material used for manufacturing the patient-specific phantom) are necessary. 4D CMR is used clinically and can generate superb imaging data, however the duration of these acquisitions still poses a major limitation for routine clinical applications. In general, the small dimensions of the anatomical structures at the time of Stage 1 palliation of HLHS can represent an additional degree of difficulty for imaging acquisitions, even when employing patient-specific phantoms.

Computational models can provide full fields of local fluid dynamics quantities [e.g., wall shear stress (59)] with the boundary conditions and model parameters straightforwardly set and reproducible (60). Implementation of compliant vessel boundaries using FSI remains in the development stages. To partly accommodate for this, multi-scale models that couple LPN models to 3D anatomical structures are an improvement over localized flow models by allowing for realistic interactions with the complete circulation. It remains crucial to demonstrate the reliability of any computational model by means of a suitable validation study.

## The Process of Decision-Making

The predictive element of engineering models could ultimately be helpful in the clinic during the decision-making process, bearing in mind the variables that can affect the success of a Norwood operation. Patient-specific virtual surgery can allow the clinician to compare different surgical options for the same child, highlighting potential differences in the local fluid dynamics and variables such as power loss and oxygen saturations. A recent study has discussed a virtual surgery application to second stage palliation of HLHS (61). With regard to the Norwood procedure, specific points that should be tackled include:

- Optimal shaping and sizing of the reconstructed aortic arch.
- Differences between shunting options (i.e., mBT, Sano, central) at a patient-specific level.
- How the previous two points affect the balance between systemic and pulmonary blood flow, as well as coronary perfusion.

Several limitations are still impeding full translation of these techniques from the bench to the bedside, in particular:

- Practical constraints: a simulation or optimization study may suggest the best solution for a specific patient, but this solution may not be feasible given the anatomical/practical constraints faced by the surgeon, e.g., optimal mBT shunt diameter may be indicated, however mBT shunt sizes are standardized and not tailored to each patient.
- Time: mounting an *in vitro* study or running a computational simulation including a patient-specific anatomical model are still time consuming for the clinical timeframe, but solutions are constantly being investigated for reducing computational costs.
- Expertise: most of the techniques discussed are still not sufficiently user-friendly for a clinical application and require the interaction between an engineer and the surgeon; while this is stimulating and enriching in a research context, it may not always be practical or feasible in a clinical context (e.g., how many centers to date have a team of biomedical engineers on site?).
- Availability of clinical data: in order to generate a patient-specific model, large multi-modality datasets are necessary (i.e., imaging data for reconstructing the anatomy together with as complete as possible hemodynamic information) and these are not always available, and may also vary depending on institutional protocols.

Nevertheless, not only these challenges are being and will be addressed, but the most immediate benefit of employing engineering tools in this context is presently represented by the fact that they generate scenarios that add to the clinician’s own intuition. Even in the context of single ventricle physiology and its surgical palliation, let us remember that a technique such as the Y graft for the Fontan baffle (62) originated in the engineering arena and is currently being assessed clinically (63).

## Future Developments

Further research involving an FSI approach, including the presence of the valves and changes in aortic arch stiffness, could be areas of interest for refining our knowledge of the Norwood physiology. The computational cost of FSI simulations can still represent a burden, although faster solutions are currently being explored (64).

Resolving potential issues related to spatial resolution and acquisition time could lead to employing 4D CMR data not only to gather additional insight into the fluid dynamics of Stage 1 circulation, but also to have a powerful tool for validation of CFD models. It is in fact important to remember that it is crucial to ensure the reliability of computational models by means of comparisons with either *in vivo* or *in vitro* data (65), and such validation process can then lead to more extensive and confident use of simulation results.

The inclusion of patient-specific tissue properties would be an additional refinement of computational models, especially for FSI simulations (66). One exciting development could be represented by taking into account the viscoelastic properties of the surrounding/supporting tissues of the arterial tree, in order to simulate more accurately the behavior of physiological tissues in FSI models (67). When simulating virtual surgery scenarios, it is also important to account for the growth of the patient and the effect of a different patient’s size on the parameters set in the model (68).

Analytical techniques such as wave intensity analysis could also be implemented in computational models as additional output parameters of interest, especially with regard to VA coupling.

## Conclusion

A range of experimental and computational models has been employed over the past two decades to improve our knowledge of palliated HLHS and to investigate the complex fluid dynamics of the Norwood physiology. These models can be further refined, at present, requiring a great effort on the engineering side to make them computationally more efficient and user-friendly for the clinicians in terms of interpreting their outputs. Thorough validation of the computational models remains mandatory, as their reliability must be strongly demonstrated prior to introducing them into the clinic. Models could be informative for devising patient-specific treatments, providing a range of virtual scenarios and evaluating the optimal hemodynamic solution, when possible. However, the engineer aiming to refine the model should always be aware of the physical constraints related to the complexities of the surgery, especially for first stage palliation of HLHS, i.e., the small dimensions of the anatomy or other concomitant complications. In other words, optimizing shunt size by a fraction of millimeter is not a feasible solution when the available conduits vary in steps of 0.5 mm, unless customized conduits were available. Therefore, this field requires a strong multidisciplinary collaboration for modeling techniques to be truly meaningful for the clinical user on the one hand, and for the clinician to provide the necessary data to set the models as accurately as possible on the other hand.

## Author Contributions

Giovanni Biglino drafted the manuscript; all authors read and revised the manuscript and approved of its content.

## Conflict of Interest Statement

The authors declare that the research was conducted in the absence of any commercial or financial relationships that could be construed as a potential conflict of interest.
